# Identification and validation of housekeeping genes in brains of the desert locust *Schistocerca gregaria *under different developmental conditions

**DOI:** 10.1186/1471-2199-10-56

**Published:** 2009-06-09

**Authors:** Matthias B Van Hiel, Pieter Van Wielendaele, Liesbet Temmerman, Sofie Van Soest, Kristel Vuerinckx, Roger Huybrechts, Jozef Vanden Broeck, Gert Simonet

**Affiliations:** 1 Animal Physiology and Neurobiology, Zoological Institute, K.U.Leuven, Naamsestraat 59, B-3000 Leuven, Belgium

## Abstract

**Background:**

To obtain reliable quantitative RT-PCR data, normalization relative to stable housekeeping genes is required. However, in practice, expression levels of 'typical' housekeeping genes have been found to vary between tissues and under different experimental conditions. To date, validation studies of reference genes in insects are extremely rare and have never been performed in locusts. In this study, putative housekeeping genes were identified in the desert locust, *Schistocerca gregaria *and two different software programs (geNorm and Normfinder) were applied to assess the stability of thesegenes.

**Results:**

We have identified seven orthologs of commonly used housekeeping genes in the desert locust. The selected genes were the orthologs of actin, *EF1a, GAPDH, RP49, TubA1, Ubi*, and *CG13220*. By employing real time RT-PCR we have analysed the expression of these housekeeping genes in brain tissue of fifth instar nymphs and adults. In the brain of fifth instar nymphs geNorm indicated *Sg-EF1a*, *Sg-GAPDH *and *Sg-RP49 *as most stable genes, while Normfinder ranked *Sg-RP49*, *Sg-EF1a *and *Sg-ACT *as most suitable candidates for normalization. The best normalization candidates for gene expression studies in the brains of adult locusts were *Sg-EF1a, Sg-GAPDH *and *Sg-Ubi *according to geNorm, while Normfinder determined *Sg-GAPDH*, *Sg-Ubi *and *Sg-ACT *as the most stable housekeeping genes.

**Conclusion:**

To perform transcript profiling studies on brains of the desert locust, the use of *Sg-RP49*, *Sg-EF1a *and *Sg-ACT *as reference genes is proposed for studies of fifth instar nymphs. In experiments with adult brains, however, the most preferred reference genes were *Sg-GAPDH*, *Sg-Ubi *and *Sg-EF1a*. These data will facilitate transcript profiling studies in desert locusts and provide a good starting point for the initial selection of genes for validation studies in other insects.

## Background

Quantitative measurements of gene expression are increasingly important in understanding biological processes and research in general. Knowledge of the expression profile of a gene can, for instance, provide evidence about its regulation and its function. With genomes of several organisms already sequenced and much more on the way, researchers are able to use this information to elucidate the transcription of a gene in a relatively straightforward manner. Using the quantitative real-time RT-PCR (qPCR) technique the expression levels of a gene can be investigated in different cells, tissues and organisms and in different conditions during development or over a preferred period of time. qPCR is also widely used to verify microarray datasets or the knockdown of a gene in RNA interference experiments and is of great value in disease diagnostics [1-3]. The analysis of qPCR data requires normalization relative to an active reference or endogenous control, which compensates for differences in sample preparation, cDNA and DNA synthesis and in the amount of the starting material. Such an internal control gene ideally has an equal transcript level in all cells at every developmental stage and is unaffected by experimental conditions. Traditionally, it is assumed that housekeeping genes (HKGs) meet these criteria, since they are necessary in every cell. Nowadays, in transcript analyses any gene with a seemingly constitutive and stable expression level is defined as a housekeeping gene. In practice, however, stable gene expression occurs only rarely and it was even suggested that such genes do not exist [4]. In line with this, it was shown that transcript levels, normalized to a single HKG, can differ more than 20-fold from the actual expression [5]. To circumvent this problem tests to validate the stability of HKGs and the use of multiple genes are supported. To this end, different software programs were developed to make a selection of housekeeping genes that are most suited for normalization [4,6,7].

Surprisingly, in almost all transcript profiling studies in insects so far, analyses to validate the choice of a set of HKGs have been overlooked. In this study we identified and examined seven HKGs in brain tissues of desert locusts (*Schistocerca gregaria*) during the last molt and the reproductive cycle. The aim of this study was to assess which of these were the most stable and therefore represent the best choice for qPCR experiments. To this end the freely available normalization programs geNorm [4] and Normfinder [6] were used. The selected genes were orthologs of the commonly used actin (*Sg-ACT*) [8] (and references therein), *RP49 *(*Sg-RP49*) [9] (and references therein), *GAPDH *(*Sg-GAPDH*), ubiquitin (*Sg-Ubi*) and *EF1a *(*Sg-EF1a*) genes, supplemented with the orthologues of *TubA1 *(*Sg-TubA1*) and *CG13220 *(*Sg-CG13220*). For the remainder of the text the prefix Sg will be omitted.

## Results

Using the database of an ongoing EST project we characterized seven housekeeping genes in the genome of *S. gregaria *(Table 1; Additional file 1); protein sequences of commonly used HKGs were blasted against the translated EST database of *S. gregaria*. In addition, *TubA1 *and *CG13220 *were selected from Flyatlas, since they showed invariant expression across the different tissues in *Drosophila melanogaster *[10]. Primers were then designed and tested (Table 2). Only primer pairs with efficiency values (E) between 95 and 105% were used. This resulted in seven primer sets for the analysis of *ACT*, *EF1a, GAPDH, RP49, TubA1, Ubi*, and *CG13220 *as potential reference genes.

**Table 1:** Name, function and sequence ID of the housekeeping genes

**HKG**	**Name of *Drosophila *orthologue**	**Flybase accession no**	**Sequence ID**	**Function**
*GAPDH*	Glyceraldehyde-3-phosphate dehydrogenase	CG8893	LC.135.C1.Contig 192	Oxidoreductase in glycolysis & gluconeogenesis
*TubA1*	*α*-tubulin 1A	CG1913	LC.572.C3.Contig 674	Cytoskeletal structure protein
*Ubi*	Ubiquitin conjugating enzyme 10	CG11624	LC.2112.C1.Contig 2271	Protein degradation
*ACT*	Actin 5C	CG4027	LC.47.C2.Contig 66	Cytoskeletal structure protein
*EF1a*	Elongation factor 1*α*	CG8280	LC.303.C1.Contig 382	Protein synthesis
*RP49*	Ribosomal protein 49	CG7939	LC.3836.C1.Contig 3963	Translation
*CG13220*	CG13220	CG13220	LC.269.C1.Contig 342	Unknown

**Table 2:** Primer sequences of housekeeping genes

**HKG**	**Forward primer**	**Reverse primer**	**Amplicon**
*GAPDH*	GTCTGATGACAACAGTGCAT	GTCCATCACGCCACAACTTTC	81
*TubA1*	TGACAATGAGGCCATCTATG	CGCAAAGATGCTGTGATTGA	118
*Ubi*	GACTTTGAGGTGTGGCGTAG	GGATCACAAACACAGAACGA	76
*ACT*	AATTACCATTGGTAACGAGCGATT	TGCTTCCATACCCAGGAATGA	73
*EF1a*	GATGCTCCAGGCCACAGAGA	TGCACAGTCGGCCTGTGAT	65
*RP49*	CGCTACAAGAAGCTTAAGAGGTCAT	CCTACGGCGCACTCTGTTG	66
*CG13220*	TGTTCAGTTTTGGCTCTGTTCTGA	ACTGTTCTCCGGCAGAATGC	62

GeNorm and Normfinder were used to evaluate the stability of the HKGs in different brain samples taken during the fifth nymphal stage and/or the adult stage. For each control gene, geNorm calculates the pairwise variation with all other control genes and the gene-stability measure, M, is defined as the average pairwise variation. Consequently, genes with a low M value have a low variation and therefore, a stable expression. Then, in a stepwise manner it eliminates the gene with the highest M value and recalculates the M value of the remaining genes, eventually yielding the two most stable genes. This is represented on a chart, showing from the left (all genes included) to the right (two genes) the mean of the M values of all remaining genes. In addition, geNorm calculates a normalization factor for every sample. Normfinder, on the other hand, uses a model-based approach. It calculates a stability value based on the intragroup variance, and includes the intergroup variance if applicable. This program selects the genes with the least expression variation over the samples and takes into account systematic differences between sample subgroups.

### Validation of housekeeping genes in fifth stage nymphs

**Figure 1:**
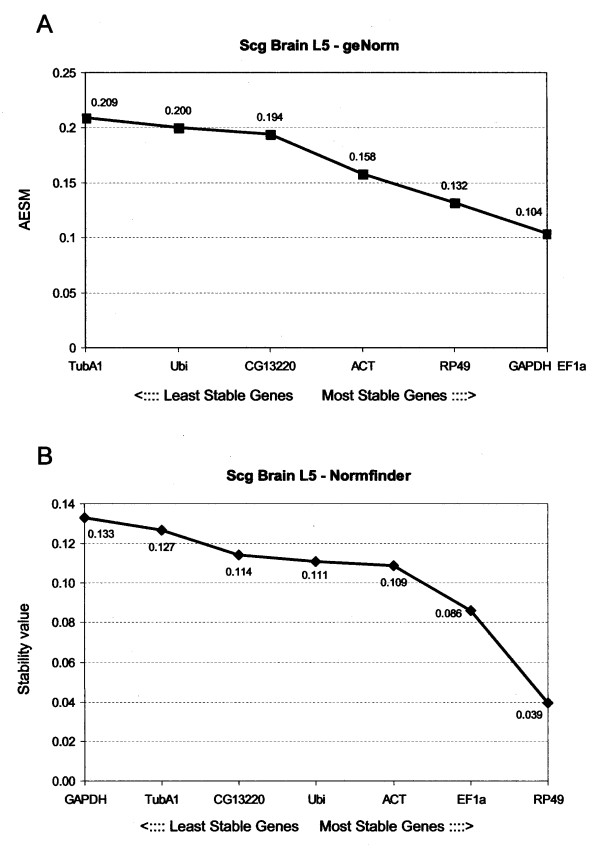
**Ranking of the housekeeping genes in desert locust brain tissues of L5 nymphs**. Gene expression stability of genes in brain tissue of L5 nymphs using two software programs; (A) geNorm gives an average expression stability measure (AESM) as the mean of the stability values of the remaining genes in a stepwise exclusion process. The lower the AESM, the more stable the gene in the subset. The threshold for an unstable gene is M ≥ 1. 5 (B) Normfinder calculates a stability value which is also inversely proportional to the stability of the gene under study.

**Figure 2:**
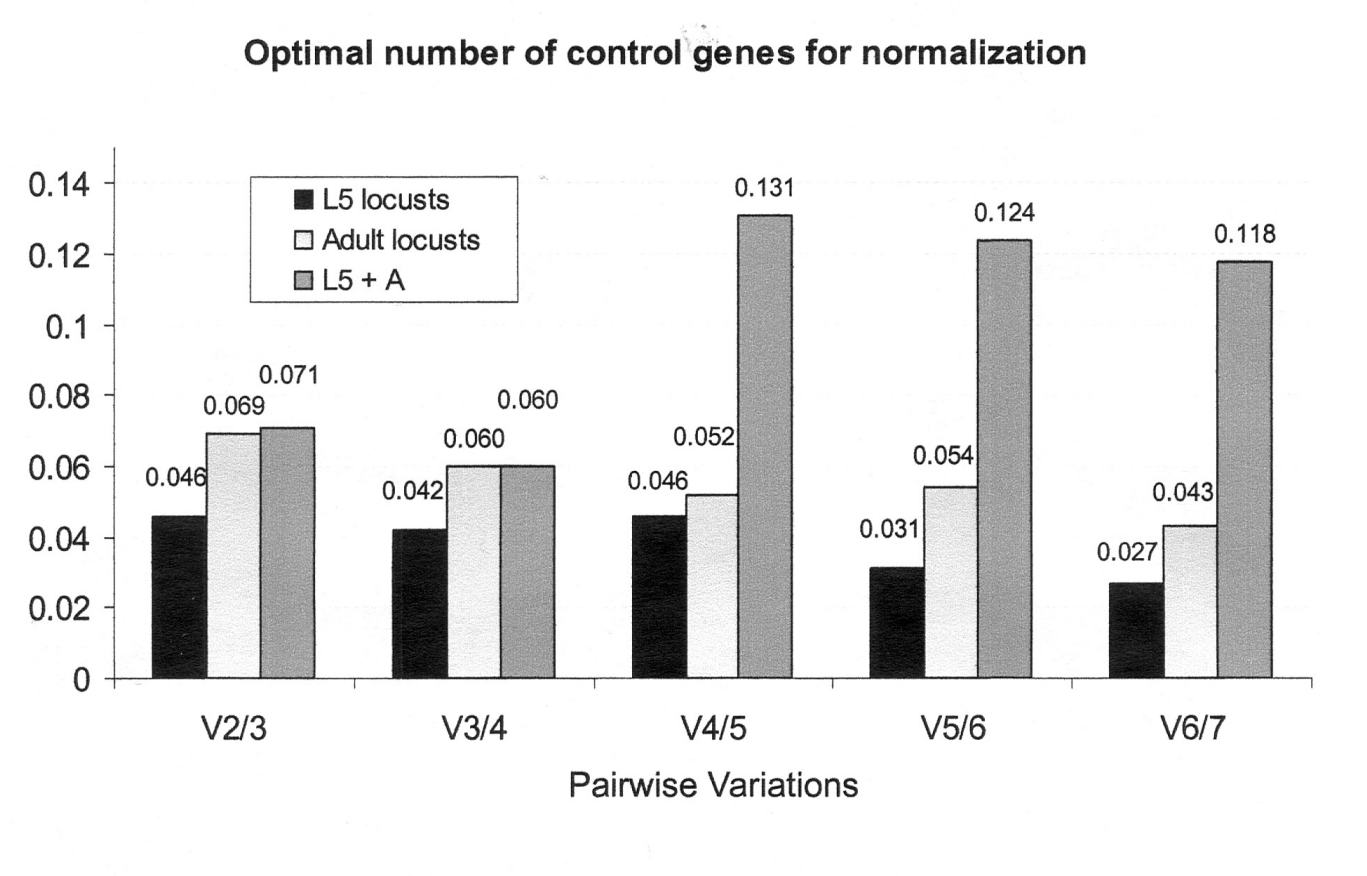
**Pairwise variation analysis**. Pairwise variation analysis (geNorm) between normalization factors NF_n _and NF_n+1 _to determine the optimal number of reference genes required for accurate normalization in *Schistocerca gregaria *for brain tissue samples of (a) L5 and (b) adult animals and (c) L5 and adult samples together.

In brain tissues from fifth stage nymphs of *Schistocerca gregaria*, geNorm indicated *EF1a *and *GAPDH *as the most stable genes, followed by *RP49 *with an average expression stability M (AESM) score of 0.104 for the combination of the first two genes and 0.132 for the combination of the three genes (Fig. 1). Normfinder ranked *RP49 *as the most stable gene (stability value of 0.039), with *EF1a *(0.086) and *ACT *(0.109) as second and third gene. This suggests that *RP49 *and *EF1a *are the best candidates for normalization. Normalisation using three genes, instead of two, is generally considered as a more robust manner to obtain a more accurate estimate of the actual transcript level of a gene of interest. Because *ACT *is ranked as a fourth gene by geNorm (AESM = 0.158) and as third gene by Normfinder, it is suggested to include this gene in normalization experiments. However, considering the low stability values, it can be concluded that the transcript levels for all of these genes are very constant. This is supported by the pairwise variation analysis by geNorm, which compares the variation (V) between two sequential normalization factors containing an increased number of genes (Fig. 2); incorporating more genes, has only little effect on the newly calculated normalisation factor. Consequently, three genes would be sufficient to accurately normalize the data, since the V_3_/V_4_-ratio is well below the cut-off of 0.15 generally accepted from Vandesompele *et al*., 2002 [4].

### Validation of housekeeping genes in adult locusts

**Figure 3:**
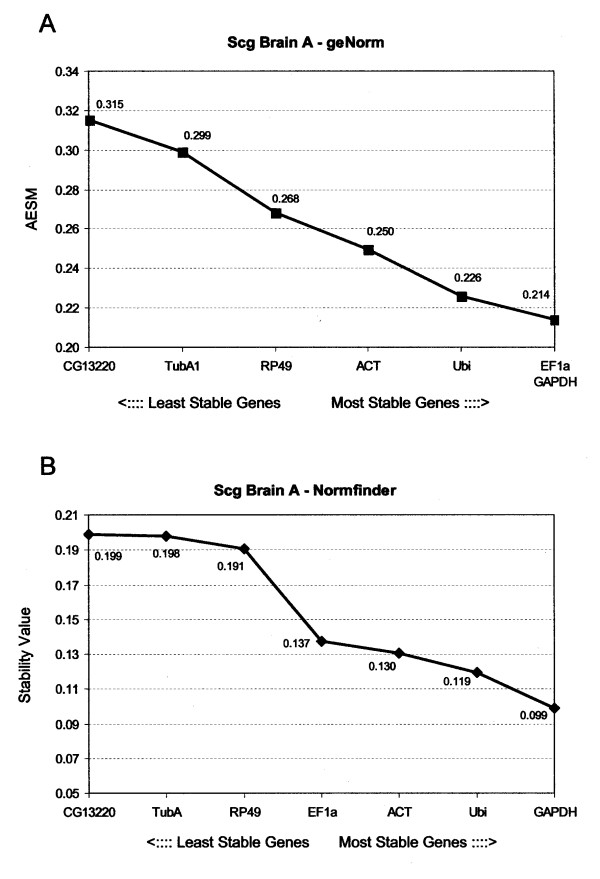
**Ranking of the housekeeping genes in adult desert locust brain tissue**. Gene expression stability of the housekeeping genes by (A) geNorm and (B) Normfinder in adult desert locust brain tissue. (A) geNorm gives an average expression stability measure (AESM) as the mean of the stability values of the remaining genes in a stepwise exclusion process. The lower the AESM, the more stable the gene in the subset. The threshold for an unstable gene is M ≥ 1. 5 (B) Normfinder calculates a stability value which is also inversely proportional to the stability of the gene under study.

The stability values for HKG transcripts in adults were generally higher than for fifth instar locusts (Fig. 3). Since M values below 1.5 are considered as acceptable, the expression of the genes can still be recognized as sufficiently stable. As in brain samples from the fifth larval stage, *EF1a *and *GAPDH *(AESM = 0.214) were ranked as best reference genes by geNorm. *Ubi *(AESM = 0.226) was ranked as third. According to Normfinder, *GAPDH *was the best choice for a reference gene in adult locusts, with a stability value of 0.099. *Ubi *(0.119) and *ACT *(0.130) were ranked as second and third genes, respectively. This implies that *GAPDH *and *Ubi *are the overall preferred reference genes. *EF1a *is ranked as best and fourth by geNorm and Normfinder (0.137) respectively. We suggest including *EF1a *as the third gene, since it has a better overall ranking than *ACT*, which was ranked as a less stable gene (fourth with a AESM of 0.250) by geNorm.

### Validation of housekeeping genes in both fifth stage nymphs and adult locusts

**Figure 4:**
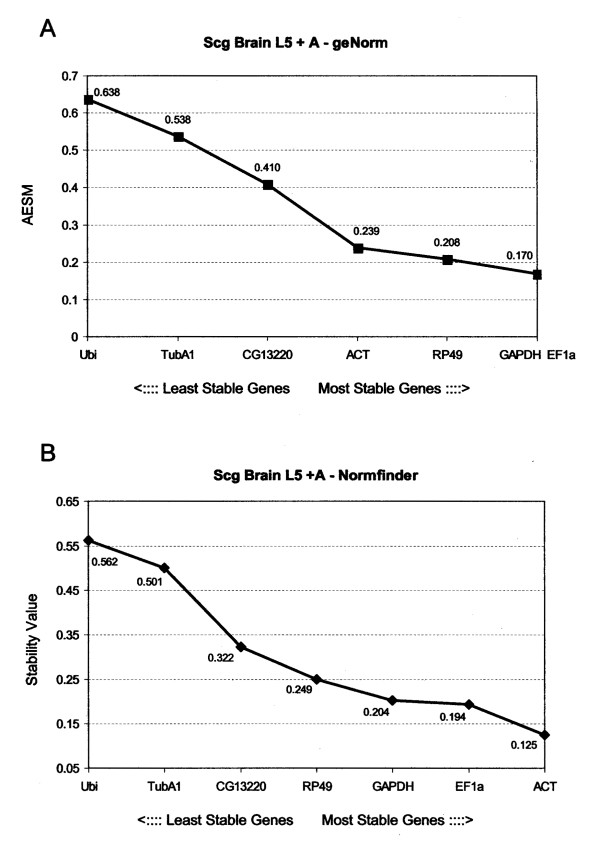
**Ranking of the housekeeping genes in desert locust brain tissues of L5 and adult specimens**. Gene expression stability of the housekeeping genes by (A) geNorm and (B) Normfinder in desert locust brain tissues of L5 and adult specimens. (A) geNorm gives an average expression stability measure (AESM) as the mean of the stability values of the remaining genes in a stepwise exclusion process. The lower the AESM, the more stable the gene in the subset. The threshold for an unstable gene is M ≥5 1. 5 (B) Normfinder calculates a stability value which is also inversely proportional to the stability of the gene in under study.

The brain tissue samples of fifth instar larvae and adult locusts were also analysed together. GeNorm indicated the same two genes (*GAPDH *and *EF1a*) as in the separate analyses as best control genes with an AESM of 0.170 (Fig 4). This is not unexpected, because these two genes were found to have the most similar expression profile in the two separate analyses (see Discussion). *RP49 *and *ACT *were ranked as third and fourth respectively. Normfinder indicated *ACT *as the best gene, with a stability score of 0.125, followed by *EF1a *(0.194), *GAPDH *(0.204) and *RP49 *(0.249). Since *ACT *has a better overall ranking than *RP49*, we propose the use of *EF1a*, *GAPDH *and *ACT *as active reference genes.

Furthermore, when the samples derived from larval and the adult animals were defined as separate subgroups in the Normfinder input, the ranking of the three best genes did not change (Additional file 2).

## Discussion

In recent years it has become clear that the accuracy of quantitative RT-PCR and microarray analyses depends strongly on the choice of the normalization genes. Numerous studies have already been performed in the search for good HKGs in a wide variety of species and tissues [11-15]. To our knowledge this is one of the very first reports on a methodical housekeeping gene analysis in insects [16,17]. In this study we identified seven putative HKGs from the desert locust *Schistocerca gregaria*. To determine the most stable genes in adult and fifth larval stage brains, samples were taken at strict time intervals covering developmental changes from the last molting cycle to the transition from juvenile to sexually mature adults and data were analyzed by two different software programs.

A first difference between the two programs is the use of a stepwise exclusion process by geNorm. This stepwise elimination of the least stable gene is helpful because of the relative nature of the determination of variability; the M value of every gene is interdependent on every other gene. This means that the ranking based on the M values (and not the AESM scores) can change after the stepwise elimination of a gene, especially when the selected genes are all relatively stable.

The geNorm principle is based on the assumption that two ideal HKGs have identical expression ratios regardless of the conditions. The software thus provides the two genes that have the most similar expression profile throughout the samples. It does this in respect to all other genes included in the survey (e.g. when two couples of similar genes are present, the two genes will be chosen that resemble the additional genes most closely). This implies, however, that co-regulated genes will always appear to be more stable. To avoid this problem we selected genes from different functional classes, as they are more likely to be independently regulated (Table 1). In addition, Normfinder was employed as an extra control. Normfinder is more resistant to the presence of co-regulated genes, because it uses a different algorithm to establish the stability of the genes. This software presents a stability value, which is directly related to the intragroup variance (when no subgroups are present) and is independent of the gene and sample. It basically calculates which gene has the smallest variation over all samples.

We applied both software programs to our data as complementary analyses to obtain the most suitable genes for our experiments. Both algorithms resulted in an overall comparable order of genes. Three of the four best genes were always presented by both programs. Peculiarly, *GAPDH *was ranked as one of the two most stable genes (together with *EF1a*) by geNorm in both tissue samples, while Normfinder ranked it as best gene in adults and as worst in fifth instars. This suggests that, while *GAPDH *and *EF1a *have a similar expression pattern (low pairwise variation) regardless of the developmental stage, the transcript levels of both genes show greater fluctuations between different brain samples (intragroup variation) from fifth instars than from adult locusts.

A third popular, freely available reference gene validation software program, termed BestKeeper [7], can also be used to analyse HKGs. However, since this program is founded on the same principle as geNorm, it was opted not to use it [18].

When comparing our results to the study of HKGs in brains of honey bees [16], it is observed that four of the genes analysed in *Apis mellifera *are putative orthologues of genes in this study (*UBQ, GAPDH, actin, αTUB*). Under the conditions used in this other study *actin*, *RPS18 *and *GAPDH *were most suited. In our experiments, *actin*, *GAPDH *and another ribosomal protein (*RP49*) were also characterized as good reference genes in either one or the other setup. Unfortunately *EF1a*, which systematically scored well in our tests, was not included in the analyses. When comparing the stability measurements of the honey bee study with ours, we can conclude that under the given conditions the genes are more unstable then in our setup. This underlines the necessity for validation of the HKGs prior to an experiment in different insect species. This is also indicated by the work of Lourenço and co-workers who tested the stability of four reference genes in different developmental stages, tissues and after an experimental treatment in the honey bee. In each case a different ranking of the genes was observed [17].

Also in locusts additional real time test experiments to validate appropriate control gene combinations should be performed when using different experimental conditions. The same is true for different tissues, tissue combinations (e.g. to measure differential expression of a gene across different tissues) or developmental stages. However, based on the identification of seven HKGs from *S. gregaria*, including a set of primer sequences to perform real-time PCR analyses, as presented in this study (Table 2), these validation tests can be performed in a relatively straightforward manner. Moreover, the genes examined in our study seem to be very stable. Even the least stable gene still has an AESM well below the cut-off of 1.5 in all three conditions, which makes this gene set a possible interesting starting point for experiments in other insects.

The desert locust is the most harmful locust species due to its ability to rapidly increase in number and to migrate over large distances [19]. This voracious insect usually exists in a harmless solitary phase, but due to changes in environmental conditions, it can transform into a gregarious phase. In this state the animals aggregate into enormous swarms comprising billions of individuals and form a threat to the food supply of millions of people. It is obvious that, when studying the behaviour of this insect, the brain is of critical importance. It is the primary neurohormone producing gland and coordinates all major processes, such as metabolism, growth and ecdysis, reproduction and behaviour. This study is important to obtain progress in our understanding of neuronal and neuro-endocrine processes at the molecular level in these insects. Foremost, it provides the right tools for the accurate elucidation of the expression profiles of genes in signaling pathways regulating developmental processes in the desert locust.

## Conclusion

We identified seven HKG in the desert locust and validated *RP49*, *EF1a *and *ACT *as the most stable genes in fifth instar nymphs and *GAPDH*, *Ubi *and *EF1a *as the most stable genes in adult locusts using the software programs geNorm and Normfinder. Overall, all tested genes proved to be rather stable, offering a good initial set of genes to be used in future validation experiments in locusts and other insects.

## Methods

### Rearing of animals

Gregarious desert locusts, *S. gregaria *(Forskål), were reared under crowded conditions with controlled temperature (32 ± 1°C), light (14 h photoperiod) and relative humidity (40–60%). The animals were kept at high density (> 200 locusts/cage) in special wooden cages and fed daily with fresh cabbage leaves and rolled oats. Mature females deposited their eggs in pots filled with slightly moistened sterile sand. After oviposition, these pots were collected once a week and set apart in empty cages, resulting in pools of hatched first instar hoppers, which differed no more than 7 days in age. Depending on the experimental conditions, locusts were further developmentally synchronized at the time of ecdysis.

### Experimental samples

For this analysis, desert locusts were synchronized immediately after the 5^th ^larval stage molt (i.e. day 0) or after the final molt. The L5 stage lasted 8 days; brains were dissected daily at the same hour (5 animals per pooled sample), yielding 8 samples. Adult brains were dissected at day 0, 4, 6, 8, 10, 12, 14, 16, 18 and 20, spanning at least 1 reproductive cycle in 10 samples (5 animals per pooled sample). Total RNA was extracted from all tissue samples, as described below, and analyzed on the ABI Prism 7000 (Applied Biosystems, Foster City, CA, USA), generating a temporal expression profile of the housekeeping gene transcripts.

### Total RNA extraction and cDNA synthesis

Locust tissues were micro-dissected under a binocular microscope and immediately collected in liquid nitrogen-cooled MagNA Lyser Green Beads (Roche, Indianapolis, IN, USA) tubes to prevent degradation. Until further processing, these pooled tissue samples were stored at -80°C. For the preparation of each total RNA sample, the pooled tissue material (≤ 20 mg) was homogenized using the MagNA Lyser instrument (Roche) according to the manufacturer's instructions. Subsequently, total RNA was extracted from the tissue homogenate utilizing the RNeasy Lipid Tissue Mini Kit (Qiagen, Valencia, CA, USA). In combination with this extraction procedure, a DNase treatment (RNase-free DNase set, Qiagen) was performed to eliminate potential genomic DNA contamination.

After spectrophotometric quantification and verification of the RNA quality via the Agilent 2100 Bioanalyser (Agilent Technologies, Palo Alto, CA, USA), the resulting total RNA was reverse transcribed (Superscript III, Invitrogen Life Technologies, Carlsbad, CA, USA) utilizing random hexamers as described in the provided protocol. To minimize variations during the cDNA synthesis step, all RNA samples were reverse transcribed simultaneously in triplicate. After cDNA synthesis the three cDNA samples from one RNA sample were mixed and 10 times diluted. Furthermore, negative control reactions, i.e. without the reverse transcriptase, were prepared and analyzed prior to the quantitative PCR assay to ascertain that no DNA contamination was present.

### Quantitative real time RT-PCR

PCR reactions were performed in a 25 μl reaction volume following the manufacturer's instructions for the SYBR Green assay (Applied Biosystems, Foster City, CA, USA). The final concentration of the primers was 300 nM. Primers for the housekeeping gene sequences of the orthologs of the *Drosophila melanogaster *genes *ACT, CG13220, EF1a, GAPDH, RP49, TubA1, Ubi *were designed by means of the Primer Express software (Applied Biosystems) (Table 2). Relative standard curves for the gene transcripts were generated with serial (5×) dilutions of brain cDNA to validate the primer sets. Efficiency of RT-PCR (E) and correlation coefficients (R^2^) were determined for each different primer pair. Reactions were run in triplicate on an ABI Prism 7000 Sequence Detection System (ABI Prism 7000 SDS, Applied Biosystems) using the following thermal cycling profile: 50°C (2 min), 95°C (10 min), followed by 40 steps of 95°C for 15 s and 60°C for 60 s. After 40 cycles, samples were run with the dissociation protocol (i.e. melting curve analysis) to check for primer dimers.

In all negative control samples no amplification of the fluorescent signal was detected, proving that the extraction procedure, including the DNase treatment, effectively removed genomic DNA from all RNA samples.

### Gene stability analysis

C_T _values for all samples were calculated (see Additional file 2) and the stability of the genes was determined utilizing two distinct algorithms: geNorm [4] and Normfinder [6].

## Authors' contributions

MV contributed in the design of the study, performed the dissections and qPCR studies in L5 locusts, executed the geNorm and Normfinder analyses and drafted the manuscript. PV performed the dissections and qPCRs in adult locust. LT provided the geNorm and Normfinder expertise and contributed in drafting the manuscript. SV performed the primer test qPCRs. KV contributed in the selection of HKGs, the design of primers and the primer test qPCRs. RH participated in the design of the study. JV contributed in the drafting of the manuscript and supervised the process. GS designed the study, selected the HKGs, supervised the process and contributed in drafting of the manuscript. All authors read and approved the final manuscript.

## Supplementary Material

Additional file 1 **Sequences of the 7 *Schistocerca gregaria *housekeeping genes**. The sequences of the identified housekeeping genes are provided in this document. The primer sequences are highlighted in black. Click here for file

Additional file 2 **Raw data of the real time PCRs**. The data provided represent the calculated average C_T _values of the locust L5 and adult brain samples. In addition, the data for both developmental conditions are represented in a graph. Click here for file
