# Supplementary material for: Identification and validation of housekeeping genes in brains of the desert locust Schistocerca gregaria under different developmental conditions
Source: BMC Mol Biol. 2009 Jun 9;10:56. doi: 10.1186/1471-2199-10-56 (PMC2700112; doi:10.1186/1471-2199-10-56)
Supplement: Additional file 1 — Sequences of the 7 Schistocerca gregaria housekeeping genes. The sequences of the identified housekeeping genes are provided in this document. The primer sequences are highlighted in black. [file 1471-2199-10-56-S1.doc]

**Identification and validation of housekeeping genes in brains of the desert locust *Schistocerca gregaria***

Matthias B. Van Hiel, Pieter Van Wielendaele, Liesbet Temmerman, Sofie Van Soest, Kristel Vuerinckx, Roger Huybrechts, Jozef Vanden Broeck, Gert Simonet

# Additional data

Additional file 1: Sequences of the 7 *Schistocerca gregaria* housekeeping genes:

Primers sequences are highlighted in black.

LC.135.C1.Contig192: Orthologue of Drm CG8893 = Gapdh2

CCGTTGCTGTCGGTTCGTAGGAAGAGGAATAGTGTCAGTGTTCGGTTCATCCAATTGTTAACAGGAAAATGTCGAAGATCGGTATCAACGGTTTTGGCCGTATTGGACGTCTGGTGCTGCGAGCAGCCCTTGACAAGGGTGCCCAGGTCGTGGCTGTTAATGATCCATTCATTGGGCTAGATTACATGGTTTACCTCTTTAAATATGACTCAACCCATGGAAAGTTCAAGGGTGAAGTGAAGGCAGATGGTGATAGTCTTGTTGTTAACGGCCACAAGATTGCAGTGTTCTCTGAACGGGACCCAAAAGATATTCCCTGGGGAAAAGCAGGTGCTGAATATATTGTAGAGTCCACTGGAGTCTTCACCACAATTGAGAAGGCCTCGGCTCACTTGGCAGGCGGTGCAAAGAAGGTTGTAATTTCTGCTCCAAGTGCAGATGCCCCAATGTATGTTGTTGGTGTAAATTTGGAAGCATATGACCCAAGTGCTAAGGTTGTTTCAAATGCTTCCTGCACTACAAATTGCCTGGCACCACTGGCAAAAGTTATCCATGACAATTTTGAAATTGTTGAAG**GTCTGATGACAACAGTGCAT**GCTGTTACTGCTACACAGAAGACTGTGGATGGGCCATCTG**GAAAGTTGTGGCGTGATGGAC**GTGGTGCTGGCCAGAACATTATTCCAGCATCAACTGGAGCTGCAAAAGCTGTTGGAAAGGTCATTCCTTCATTGAATGGGAAACTCACTGGAATGGCTTTTAGAGTCCCAGTGCCAAATGTTTCAGTTGTTGACCTCACTGTCAGGTTGGGCAAGGGTGCCAGCTACGATGATATCAAGGCCAAGGTGAAGGAAGCTGCTGAAGGTCCTTTGAAAGGAATCTTGGCATATACTGAGGATGACGTTGTGTCTTCAGATTTCATTGGTGATTGCCATTCTTCAATCTTTGATGCTAAGGCAGGCATCCCACTCAACAATAACTTTGTTAAGCTTGTTTCCTGGTATGACAATGAATTTGGTTACTCAAACCGTGTTGTGGACCTGATAAAGTTCATGCAGTCAAAGGACTAAATTGTTCCATGTGCTACAGTTCATAATGTTGGTGTGATCAGCAGTCCTATGACCCCCCACAAAACTGAATGTGTCAGATCAGTATTGCAGTGATCAGTGGTGTTATTCCATAATTGTGACTGATTCATGGCAGTGTTTCTGCTTAATAGCTAGGCTCTAGGAATTTGATAGAAAGTTGAAAAATATTGATTTAGAATTAATATAATCAAAAGCTTTGTTAAATTGTAAACATACTCATTGAACCTAGTCATGAGTCTTCAGTTCAAAAGTTGAATAAATGCTGATGATATTGCTGTTGAAAAAAAAAAAAAAAAAATCGGT

LC.572.C2.Contig673: Orthologue of Drm CG1913 = alphaTub84B

AGGGGACTGTCCGTTTGGGAAAAGCGTCGTGGTTGCTGCTCCTGAACGACTGGGTTGTTGTAACGGTTTTTTTGTGTGTGACTAGTGTGTTGAATATTTACCATGCGTGAATGCATCTCTATTCATGTTGGCCAAGCCGGCGTACAGATCGGGAACGCGTGCTGGGAATTGTACTGCCTGGAACATGGAATTCAGCCTGATGGGCAGATGCCCTCAGACAAAACAATTGGCGGAGGAGACGATAGTTTCAACACATTCTTTAGCGAAACGGGCGCAGGAAAACATGTACCTAGGGCAGTTTTCGTAGACCTGGAACCCACCGTTGTGGATGAGGTACGCACTGGCACCTATCGGCAGCTGTTTCACCCTGAGCAGCTCATCACTGGCAAGGAGGATGCTGCAAACAATTATGCACGTGGTCACTATACAATTGGAAAAGAGATTGTGGACTTGGTGTTGGATCGCATCAGGAAGTTAGCGGATCAGTGTACTGGGTTGCAAGGGTTCCTTATTTTCCACTCTTTCGGCGGTGGCACTGGATCTGGCTTCACATCATTGCTTATGGAACGATTATCGGTAGACTATGGCAAGAAGAGCAAACTGGAATTTGCAATCTATCCAGCACCCCAAGTTTCTACTGCTGTTGTAGAACCATATAATTCCATCCTTACAACTCACACTACCCTCGAACATTCAGATTGTGCTTTCATGGT**TGACAATGAGGCCATCTATG**ACATCTGTCGCCGAAACCTGGACATTGAACGCCCTACATACACAAATCTTAACAGATTAATTGGTCAGATTGTCTCC**TCAATCACAGCATCTTTGCG**TTTTGATGGTGCACTCAATGTTGACCTGACTGAGTTCCAAACTAAtcaaCCTTGTGCCCTACCCCCGTATCCATTTTCCACTTGTCACGTATGCTCCTGTCATATCGGCAGAAAAGGCATACCATGAACAACTATCAGTTGCAGAGATTACAAATGCTTGCTTTGAGCCAGCCAATCAGATGGTAAAATGTGACCCAAGGCATGGAAAGTACATGGCTTGCTGCATGCTTTACAGAGGTGATGTCGTTCCTAAGGATGTCAATGCTGCCATTGCTACCATTAAGACGAAGCGAACAATTCAGTTTGTTGATTGGTGTCCCACAGGTTTCAAGGTCGGAATAAATTACCAGCCTCCTACTGTTGTTCCTGGAGGCGATTTAGCAAAGGTTCAGAGAGCTGTGTGCATGTTATCAAACACAACTGCTATTGCTGAAGCTTGGGCCCGACTTGATCACAAGTTCGATCTCATGTATGCCAAACGTGCATTTGTCCATTGGTATGTTGGTGAGGGTATGGAAGAAGGGGAGTTCTCGGAGGCCAGAGAAGATTTGGCTGCCCTTGAAAAAGATTACGAGGAGGTGGGAATGGATTCAGTTGAAGGAGAAGGCGAAGGTGCTGAGGAGTACTGAGTGCTTAAAATGTCATTTGAATTGTATTTATACTTTTGTATACCAGGAGATCATTCCAAGTTTTGGTAAATGTATCATTTTCAGAACTGAATGAATTGCATTGAAGCATATTTATGTGTATTCATTGATTATATTTCCTGAAAGTGCCACCTCCATAAACGTGCTCAGTTTTAAGTAGACAGTAAAACTAAACAATCTGAATAACAGTTGCAATTGGATCCTGGCCTNNNTAATAAGTTTTAACCCCTACATTTTTTAAAGCAAATGTTCACATGAAAGGAACCTACTTTTCAAGAAAGTGCTAAGTGCT

LC.2112.C1.Contig2271: Orthologue of Drm CG11624 = Ubi-p63E

NCACACCATTTAAGCGAAGTTGGGACTGTCATTGTCATAC**GACTTTGAGGTGTGGCGTAG**CTGGTTGAAATTTTGACTGTAGTTCGCTTTTGACAG**TCGTTCTGTGTTTGTGATCC**CACAAAGCAGATATGCAGATCTTTGTAAAAACATTAACAGGGAAGACAATAACACTTGAAGTTGAACCTTCAGATACAATAGAAAATGTTAAAGCAAAGATTCAAGACAAAGAAGGCATTCCACCTGATCAGCAACGTTTGATATTTGCTGGTAAACAGCTGGAAGATGGCCGCACTTTGTCAGATTACAACATACAAAAAGAATCAACACTTCACTTGGTTCTCCGTCTCAGGGGTGGGATGCAAATATTTGTCAAAACTTTGACTGGCAAAACAATAACACTGGAAGTTGAGCCTTCGGACACAATAGAAAATGTAAAGGCAAAAATTCAGGATAAAGAAGGCATTCCACCAGACCAGCAGAGGCTAATTTTTGCTGGAAAACAGTTGGAAGATGGACGTACACTGTCAGATTACAATATTCAAAAAGAATCTACATTGCATCTAGTGTTACGTCTTAGAGGAGGCATGCAGATTTTTGTAAAAACTCTGACTGGGAAAACAATAACGTTGGAAGTTGAACCATCAGACACAATTGAAAACGTAAAAGCAAAAATCCAAGACAAGGAAGGGATTCCACCAGACCAACAGAGATTAATTTTTGCTGGGAAACAGCTTGAAGATGGAAGAACTCTCTCAGATTACAACATACAAAAAGAATCCACATTGCATTTGGTGTTGCGTCTTAGAGGTGGCATGCAGATATTTGTAAAAACTCTGACTGGGAAAACAATAACATTGGAAGTGGAGCCATCAGACACAATTGAAAATGTAAAAGCAAAAATTCAAGACAAGGAAGGGATTCCACCAGACCAACAGAGATTAATCTTTGCTGGAAAACAGCTTGAAGATGGAAGAACTCTCTCAGATTACAACATACAAAAAGAATCAACTTTGCATCTGGTGTTGCGTCTGCGTGGTGGTATGCAAATTTTTGTAAAAACTCTTACTGGCAAAACAATAACTCTAGAAGTGGAGCCATCAGATACCATTGAAAATGTAAAGGCAAAAATTCAGGATAAAGAAGGGATCCCACCAGATCAACAGAGACTCATCTTTGCAGGGAAACAACTAGAAGATGG

LC.47.C2.Contig66: Orthologue of Drm CG4027 = Act5C

NCTGTCGCCGTTGCTGTCGGTTTGTTGTGGAGTACGGAAGGTGTGTGCAGTGGTCTCCGACAAGTTCCAATAATTCAACATGTGTGACGAAGAAGTTGCTGCTCTCGTTGTGGACAATGGATCCGGAATGTGCAAGGCTGGCTTCGCCGGCGATGATGCACCAAGGGCCGTATTCCCTTCAATTGTTGGTCGCCCAAGGCATCAGGGAGTGATGGTGGGCATGGGACAGAAAGATAGCTATGTTGGTGACGAAGCCCAGTCTAAGAGAGGTATCCTTACTCTGAAGTATCCAATTGAGCACGGAATTGTCACAAACTGGGACGACATGGAAAAAATTTGGCACCACACCTTCTACAACGAGCTTAGGGTGGCGCCCGAGGAACATCCCGTTCTTCTGACTGAAGCCCCATTGAACCCCAAGGCAAACAGGGAGAAGATGACTCAGATCATGTTCGAAACTTTCAACACCCCAGCCATGTACGTAGCTATTCAGGCCGTGCTCTCCCTGTACGCCTCTGGTAGGACAACTGGTATTGTGTTGGATTCTGGTGATGGCGTTTCGCACACCGTGCCAATTTATGAAGGTTATGCCCTGCCCCATGCCATCCTCAGATTGGACTTGGCTGGCCGTGATTTAACAGACTACCTGATGAAGATTCTTACTGAGAGGGGCTACAGCTTTACCACCACAGCTGAACGAGAAATTGTAAGAGACATTAAGGAGAAATTGTGCTATGTTGCACTAGACTTTGAACAGGAGATGGCTACTGCTGCATCCTCCAGTTCTCTAGAGAAATCCTATGAATTGCCTGATGGCCAGGT**AATTACCATTGGTAACGAGCGATT**CAGGTGCCCTGAGGCACTGTTCCAGCCT**TCATTCCTGGGTATGGAAGCA**AATGGTATCCACGAAACTACCTACAACTCTATCATGAAGTGTGATGTAGACATCAGGAAAGATCTGTATGCCAACACAGTACTTTCTGGTGGTACTACCATGTACCCTGGCATTGCTGACAGGATGCAGAAGGAAATCACTGCCCTTGCACCTTCTACCATGAAGATAAAGATCATTGCTCCACCTGAGAGGAAGTACTCAGTCTGGATTGGAGGTTCCATCTTGGCATCTCTTTCCACCTTCCAGCAGATGTGGATCTCTAAGCAAGAATATGATGAATCTGGTCCATCAATTGTCCACAGGAAATGCTTCTAAGCAGTTGCCTAATTATTTTGTTCAGGTGAAGTATTTATACTTCTATCATGCCAGACCATTTCAGGCACCACCAGGTGTGGCCAAAAGACTTTCTTTATTGTATTATGTTGAACAGTTTAAACTCCTTGTATTATAAGATTCGAAGTGTTTTCGTTACAATTTACAATTGAGGAAGGTAGGCTGCATTTTTCTATTTAAGAATCACCTCATTATATCCTGGTGTTTCTGTGACTGAGTAACTGAAATGTGATGTATAACTTGTGTTAAAATTGAAGAAAGTGGATAAGCTTCCTGTACCAGAATGTAAATTTCTTAATGTTTTGTATTTGAAATGACTTTTGTATTGCATTTATTAAGCAATTCACAAGTTGTGCAAGTTTCCTTTCTAAGCAACTTGGCATTGTCTTGAGATTAAAGATGTGCTGAGTAAATGTCAATAGCCAAAGTATTAACTTATTCCCATGTGCAGACAAGAAATTAGTGGGAAAATAAAACTTATTTAAAAAAAAAAAAAAAAAAAAAAAAAAAAAAAATGC

LC.303.C1.Contig382: Orthologue of Drm CG8280 = Ef1alpha48D

GGACTGTCTGCAGTGTTCTGCGTATCGGGTGGATATCACAGTAAAGTTATTAGACTGAAGAACTCCCTGTAAACTACAAAGATGGGTAAGGAAAAGATTCACATTAACATCGTCGTCATTGGTCACGTAGATTCCGGCAAATCGACCACGACCGGACATTTGATCTACAAATGTGGTGGAATAGACAAGAGAACCATCGAAAAATTCGAAAAGGAAGCCCAGGAGATGGGCAAGGGTTCGTTCAAGTATGCCTGGGTGTTGGATAAGCTGAAGGCAGAACGTGAACGTGGTATCACGATTGACATTGCTTTATGGAAGTTCGAAACTAGCAAGTACTACGTGACCATCATA**GATGCTCCAGGCCACAGAGA**TTTCATTAAGAACATGATTACAGGAAC**ATCACAGGCCGACTGTGCA**GTGTTGATTGTAGCAGCTGGTACAGGTGAATTTGAAGCCGGTATTTCGAAGAACGGTCAGACTCGTGAGCACGCACTGTTGGCATTCACTTTGGGTGTGAAACAACTGATCGTGGGTGTGAACAAAATGGACTCGACTGAGCCACCATACAGTGAGGCTAGGTTTGAGGAAATTAAGAAGGAAGTAAGCAATTACATTAAGAAGATTGGTTACAATCCTGCAGCTGTTGCCTTTGTCCCAATTTCCGGATGGCATGGCGACAACATGCTTGAGCATTCTGACAAGATGGGCTGGTTCAAGGGATGGTCTATTGAGCGTAAGGAAGGGAAGGCCGAGGGGAAGACCTTAATTGAAGCTCTCGATGCTATCCTTCCTCCTAGCAGACCAACTGAAAAGCCTCTG

LC.3836.C1.Contig3963: Orthologue of Drm CG7939 = RpL32 = Rp49

GTGTGGCGGATGAGAAGTTGGAGAGAAAAATAAGTGTACTTGGTGACAATAATTATATAGATAATGGCGATTCGACCAGTGTATAGACCAACTATTGTCAAGAAGAGGACGAAACACTTCATTCGTCATCAGAGCGAT**CGCTACAAGAAGCTTAAGAGGTCAT**GGCGTAAACCAAAGGGAATTGA**CAACAGAGTGCGCCGTAGG**TTTAAGGGCCAGTACCTTATGCCATCCATTGGTTATGGTAGCAGTCGTAAGACAAAGCATATGCTACCAACAGGATTCCGCAAGGTTTTGGTACATAATGTCAGGGAACTGGAAGTCTTGATGATGCAGAACCGCAAATTTTGTGCTGAAATTGCACATGCGGTTTCTTCAAAGAAACGTAAAGCTATTGTGGAGCGGGCACAGCAGCTATCCATCAGAGTGACCAATGCTAATGCCCGCTTACGCAGTGAAGAAAATGAATAATGTGACTTATCATTGTAATAAAATAAAATTCTCAAATATTTTATAACTTGTTCAGATACATTCTTTAATGTTACCTCCCACTCACCTTTGGTTTTGTTTACAGTATTGAATAACTACTATGTTTTTTTCATATCTGATCTGTTAAGAAAAGTGTCTAAACTTCACCAGATTTTTTCCATCGAATTACTTGTAAAAGAGAATTGATTAACTTGTTTGCTGGTGTTAGCACACAGAGCCACAAGAATTGTATTTTGCTTTATAATTGCGAATGGCATACATGCAAATTTC

LC.269.C1.Contig342: no annotation available

CTGTCGGTGAAAATCGATGTACGCTATTTCTTCTGTTCTATTTATGTTTGCTCAAACAGTGTAAAATTTTTGTCAACACCATGGATTCAGCATTCAAAAGGGACAAGGATAACAAGAAGACAGTGGTGTCAAAATTTGAGTTATTTAAGAAACCCATCAACACTCATAATGTACTGTTTTACTACGTTCCATTCTACGGAGCAGCGAGTTACAGTGCTTTATCTGTTAATGTTTTAAATCCCGGCTTGGGAGTAAGGATGTTCCCGAAACGTGATGTTACAAATATATTGCTGTTGTCTACGTTAATTGGAACTGGTCTGTATATATATGACAGGAAGCACATGAGAGCAGCACCGAAAATTCCAAAAGTTGGATACAGTGTATTTGGAGCTCTAA**TGTTCAGTTTTGGCTCTGTTCTGA**TGTGGGCAGTAATGAGGA**GCATTCTGCCGGAGAACAGT**ATTATTGCTACAACCTGTGGCATATCGTCAGCACTTGGATTAGTAAAAGCTGGAACAAATTATTTGGAGTTTGTTGACTCACAGGTGAAAAACTGAGTTTTTCTTATGAAAGTACCCTTTGTATGTTATGAATTTTCGGAGTACAATTAGATAATTTAGCTTCAATGTAAAGGAGTCACCAATTTCATTATGTGTTACCATATAATATTTACTGTTATGTAAATTTAAAAGTGCCAAGCATTTTTCCCAGATACAAATTCTTAACTGTAGTCACTTTGATCAATGACTGCACCCTTATTACAAATGTATTTCTTATTAAAGAGGCATGCTGATTACACCTCAGAAAGCAACAGGATATTTTTCTGTGTATATTTCCTTTGTGTATTTTGTTGTGCTTTCCAATTTTTAAATATTGAGGAATGTTGTTTCTGGCAGTCACCCTCATATTTTTGTTTGTTGAAACTGGTTTGTAACAACCTTTAAAAAAAAAAAAAAATACATCGTTGAATATGTTATATACCCAGGAAGGATATCTCCATTTTGAATATGTATTTTGTAAGTGTACAACCACTATTAGCCTGAAATAAATGAAATAAAATGTTTTGATGTGTAAAAAAAAAAAAAAAAAATCGGT
