# Supplementary material for: Identification and validation of housekeeping genes in brains of the desert locust Schistocerca gregaria under different developmental conditions
Source: BMC Mol Biol. 2009 Jun 9;10:56. doi: 10.1186/1471-2199-10-56 (PMC2700112; doi:10.1186/1471-2199-10-56)
Supplement: Additional file 2 — Raw data of the real time PCRs. The data provided represent the calculated average CT values of the locust L5 and adult brain samples. In addition, the data for both developmental conditions are represented in a graph. [file 1471-2199-10-56-S2.doc]

**Identification and validation of housekeeping genes in brains of the desert locust *Schistocerca gregaria***

Matthias B. Van Hiel, Pieter Van Wielendaele, Liesbet Temmerman, Sofie Van Soest, Kristel Vuerinckx, Roger Huybrechts, Jozef Vanden Broeck, Gert Simonet

**Additional file 2:**

Additional Table 1: C_T_ values Scg-L5 brains:

|  | ACT | GAPDH | TubA1 | Ubi | EF1a | RP49 | CG13220 |
| --- | --- | --- | --- | --- | --- | --- | --- |
| L5Brd1 | 19,54448 | 20,5159 | 21,7967 | 20,9152 | 18,29695 | 20,9641 | 22,4297 |
| L5Brd2 | 19,8894 | 20,42165 | 22,301 | 21,24105 | 18,47745 | 21,24855 | 22,91315 |
| L5Brd3 | 19,19285 | 19,73605 | 21,75235 | 20,67585 | 17,66735 | 20,58475 | 22,13375 |
| L5Brd4 | 19,24945 | 19,8565 | 22,00205 | 20,9029 | 17,76215 | 20,7697 | 22,3104 |
| L5Brd5 | 19,81865 | 20,23415 | 22,27925 | 21,68035 | 18,2326 | 21,10195 | 23,24045 |
| L5Brd6 | 19,62578 | 20,25505 | 22,1891 | 21,32485 | 18,15985 | 21,08625 | 22,8023 |
| L5Brd7 | 19,37308 | 20,1344 | 22,2859 | 21,3312 | 18,19155 | 21,1889 | 22,82255 |
| L5Brd8 | 19,81008 | 20,2281 | 21,8236 | 21,16165 | 18,0333 | 21,03045 | 22,6863 |

Additional Figure 1: Distribution of C_T_ values of HKGs in Scg brain samples of L5 nymphs

Additional Table 2: Ct values Scg A brains:

|  | ACT | GAPDH | TubA1 | Ubi | EF1a | RP49 | CG13220 |
| --- | --- | --- | --- | --- | --- | --- | --- |
| Ad0 | 18,94235 | 19,632 | 20,92843 | 22,2811 | 17,58937 | 20,37742 | 23,0413 |
| Ad4 | 19,37797 | 19,88465 | 22,26877 | 22,96005 | 18,00517 | 20,46099 | 23,42593 |
| Ad6 | 18,9827 | 19,0294 | 21,46163 | 22,24075 | 17,22593 | 20,09349 | 23,15033 |
| Ad8 | 19,99763 | 19,92303 | 21,77813 | 22,8395 | 17,86813 | 20,69238 | 23,5165 |
| Ad10 | 19,95483 | 20,4419 | 21,85727 | 23,24797 | 18,43153 | 21,11342 | 23,8826 |
| Ad12 | 20,26337 | 20,37723 | 22,06357 | 22,953 | 18,66467 | 21,34385 | 23,76797 |
| Ad14 | 19,47313 | 19,83177 | 21,61347 | 22,41693 | 17,5709 | 20,34293 | 23,83605 |
| Ad16 | 19,36877 | 19,70993 | 21,68217 | 22,33277 | 17,3653 | 20,46382 | 23,81665 |
| Ad18 | 20,8479 | 21,47867 | 23,33883 | 24,38917 | 19,15403 | 21,48402 | 25,1991 |
| Ad20 | 20,045 | 20,55407 | 22,4677 | 23,38965 | 18,5188 | 21,55062 | 24,0352 |

Additional Figure 2: Distribution of C_T_ values of HKGs in Scg brain samples of adults

Additional Table 3: Normfinder analyses of the *S. gregaria* brain samples of L5 nymphs and adults with or without using subgroups (with or without intergroup variation).

| **w/o subgroups** | | **w/ subgroups** | |
| --- | --- | --- | --- |
| **Gene name** | **Stability value** | **Gene name** | **Stability value** |
| ACT | 0,125 | ACT | 0,146 |
| EF1a | 0,194 | EF1a | 0,212 |
| GAPDH | 0,204 | GAPDH | 0,223 |
| RP49 | 0,249 | TubA1 | 0,239 |
| CG13220 | 0,322 | Rp49 | 0,250 |
| TubA1 | 0,501 | CG13220 | 0,313 |
| Ubi | 0,562 | Ubi | 0,514 |
